# Integration of histopathological image features and multi-dimensional omics data in predicting molecular features and survival in glioblastoma

**DOI:** 10.3389/fmed.2025.1510793

**Published:** 2025-04-23

**Authors:** Yeqian Huang, Linyan Chen, Zhiyuan Zhang, Yu Liu, Leizhen Huang, Yang Liu, Pengcheng Liu, Fengqin Song, Zhengyong Li, Zhenyu Zhang

**Affiliations:** ^1^Department of Burn and Plastic Surgery, West China Hospital, Sichuan University, Chengdu, China; ^2^Department of Biotherapy, Cancer Center and State Key Laboratory of Biotherapy, West China Hospital, Sichuan University, Chengdu, China; ^3^West China School of Medicine, West China Hospital, Sichuan University, Chengdu, China; ^4^Department of Plastic Reconstructive and Aesthetic Surgery, West China Tianfu Hospital, Sichuan University, Chengdu, China

**Keywords:** glioblastoma, histopathological image, genomics, transcriptomics, proteomics, prognosis

## Abstract

**Objectives:**

Glioblastoma (GBM) is a highly malignant brain tumor with complex molecular mechanisms. Histopathological images provide valuable morphological information of tumors. This study aims to evaluate the predictive potential of quantitative histopathological image features (HIF) for molecular characteristics and overall survival (OS) in GBM patients by integrating HIF with multi-omics data.

**Methods:**

We included 439 GBM patients with eligible histopathological images and corresponding genetic data from The Cancer Genome Atlas (TCGA). A total of 550 image features were extracted from the histopathological images. Machine learning algorithms were employed to identify molecular characteristics, with random forest (RF) models demonstrating the best predictive performance. Predictive models for OS were constructed based on HIF using RF. Additionally, we enrolled tissue microarrays of 67 patients as an external validation set. The prognostic histopathological image features (PHIF) were identified using two machine learning algorithms, and prognosis-related gene modules were discovered through WGCNA.

**Results:**

The RF-based OS prediction model achieved significant prognostic accuracy (5-year AUC = 0.829). Prognostic models were also developed using single-omics, the integration of HIF and single-omics (HIF + genomics, HIF + transcriptomics, HIF + proteomics), and all features (multi-omics). The multi-omics model achieved the best prediction performance (1-, 3- and 5-year AUCs of 0.820, 0.926 and 0.878, respectively).

**Conclusion:**

Our study indicated a certain prognostic value of HIF, and the integrated multi-omics model may enhance the prognostic prediction of GBM, offering improved accuracy and robustness for clinical application.

## 1 Introduction

Glioma is the most prevalent primary malignant tumor of the brain, accounting for 40–50% of intracranial tumors (1). Glioblastoma (GBM), classified as a WHO grade IV glioma, is the most common (57.3% of all gliomas) and aggressive form of glioma in adults (2, 3). The age-adjusted incidence rate of GBM is 3.22 per 100,000 population, with a median overall survival (OS) of 12–15 months with standard treatment, while population studies suggest a median survival of 8–10 months (4, 5). Approximately 7% of GBM patients live for at least 5 years after diagnosis, defined as long-term survivors (LTS) in previous research (6–8). Conventional treatments of GBM include maximal surgical resection, postoperative radiotherapy and chemotherapy; however, complete tumor resection is often unattainable due to the tumor’s invasive nature and high recurrence rate (9). Prognostic factors such as tumor stage, age, pathological grade, KPS, extent of resection and certain molecular markers have been identified as key indicators of GBM prognosis (10, 11). Therefore, as a cancer characterized by multiple genetic and pathway alterations, further investigation into comprehensive prognostic markers is critical for guiding risk stratification, clinical treatment decisions and survival prediction in GBM patients.

GBM derives from glial cells and neurons and exhibits a complex gene expression profile with various molecular alterations that drive its oncogenesis and progression (12). Notably, isocitrate dehydrogenase-1 (IDH-1) and IDH-2 mutations are observed in primary (6%) and secondary (70%) GBMs (13). Compared with IDH1 wild-type, the survival of IDH1 mutant high-grade glioma patients is significantly prolonged (14). The O6-methylguanine-DNA methyltransferase (MGMT) coded protein involved in methylated bases and DNA repair and the methylation status of MGMT promoter may be a significant predictor for sensitivity to chemotherapy or radiotherapy (15, 16). Telomerase reverse transcriptase (TERT) can activate telomerase to keep the telomeres intact and promote cell proliferation. IDH1 mutant gliomas with mutations in TERT promoter have exhibited better prognosis (17). Alpha thalassemia/X-linked intellectual disability (ATRX) is also discovered as a mutational cancer driver in GBM (18). GBM can be classified into subtypes based on molecular features, including transcriptional profiles (classical, mesenchymal, neural, proneural), genetic mutations (e.g., IDH1 mutations), and epigenetic alterations (e.g., CpG island methylator phenotype, CIMP) and so on (19, 20). Therefore, establishing a comprehensive and effective biomarker will be of great benefit to prognostic prediction and therapeutic strategies for GBM patients.

In clinical practice, in addition to imaging examinations such as CT and MRI, the final diagnosis is confirmed through histopathological biopsy following tumor resection. Histopathological images obtained from H&E-stained tumor tissue slides are routinely used in definite diagnosis and staging of different cancers. The development of computer-assisted medical image processing and analysis systems is increasingly employed in digital pathological image assessment. These systems can accurately and reproducibly capture morphological, structural, and compositional changes in tissues and cells, reducing the subjectivity associated with traditional pathologist assessments (21). Commonly extracted histopathological image features such as texture structure, gray level distribution and morphological features including the size and shape of cell and nuclei, have demonstrated potential in pathological diagnosis, classification and prognosis of human cancers such as breast cancer (22), colorectal cancer (23) and lung cancer (24). In addition to histopathological images, omics profiles such as genomics, transcriptomics and proteomics have also been applied to patient stratification and prognostic prediction. Integrating histopathological image features with multi-omics data has shown promise in various cancers, including renal cancer (25), lung cancer (26) and head and neck squamous cell carcinoma (27). Therefore, exploring the integration of histopathological image features with omics data holds significant potential for prognostic prediction in clinical settings.

In this study, we focused on the analyses of histopathological image features (HIF) and their correlation with genomic and transcriptomic profiles, which has not been explicitly demonstrated in GBM. We first assessed the overall capacity of HIF in classifying somatic mutations, molecular and methylation subtypes of GBM via different machine learning approaches. Subsequently, we identified the prognosis-related histopathological image features and evaluated the underlying correlation with gene expression profiles. Finally, we constructed survival prediction models based on various omics profiles and their integration. We validate these models with both an internal test cohort and an external validation cohort, expecting to enhance the accuracy of prognostic prediction for GBM patients.

## 2 Materials and methods

### 2.1 Study design and data acquisition

**Figure 1:**
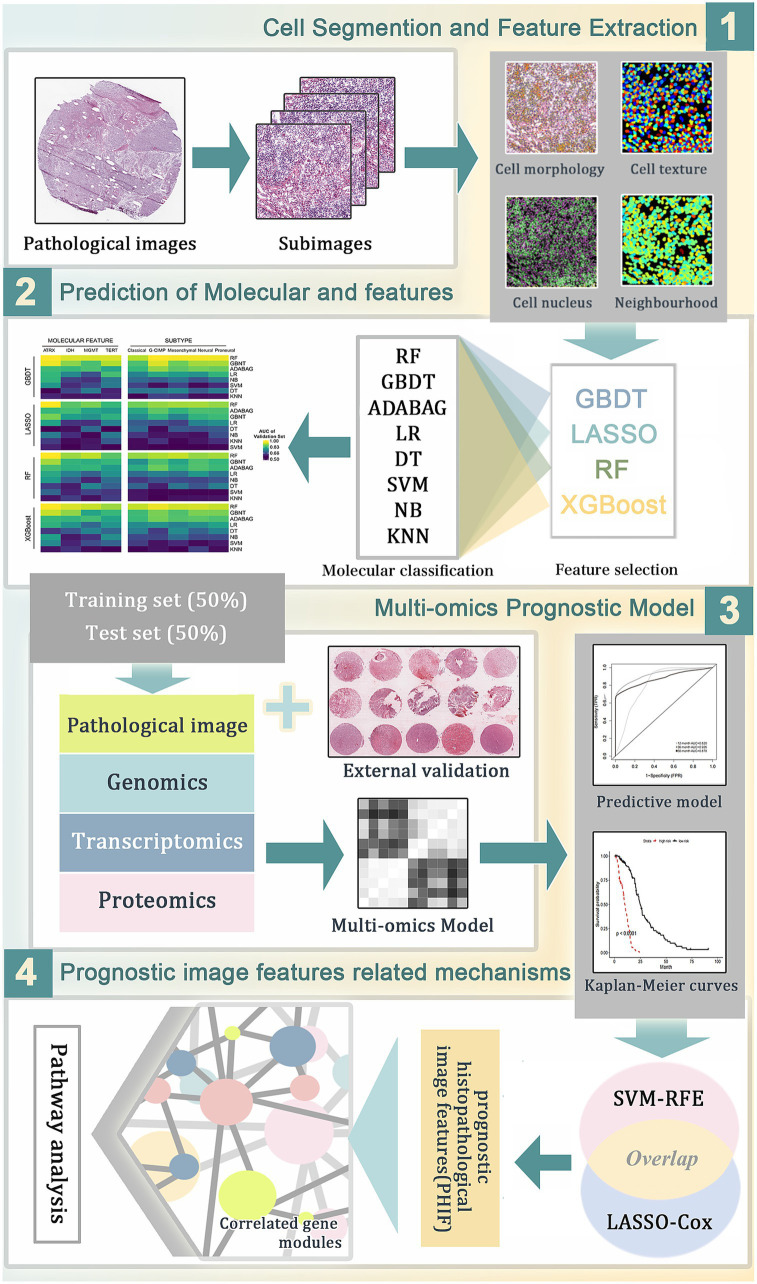
The workflow of data analysis and prognostic model construction. (1) The whole-slide histopathological images of GBM were segmented into sub-images of 1,000 × 1,000 pixels. Through CellProfiler the histopathological image features (HIF) were extracted for subsequent analyses. (2) Image feature selection and molecular features prediction based on HIF using different combinations of machine learning algorithms. (3) Construction of prognostic models for overall survival in TCGA training set based on HIF genomics, transcriptomics and proteomics data. (4) Selection of prognostic histopathological image features (PHIF) by two machine learning methods. Identification of prognostic gene modules and gene pathway analysis were performed subsequently.

The overall framework of the study is illustrated in Figure 1, and the specific process is described in the following sections. We obtained a cohort of GBM samples with accessible clinical information, genomics and transcriptomics data from The Cancer Genome Atlas (TCGA) data portal^1^ and matched proteomics profile from The Cancer Proteome Atlas (TCPA) repository.^2^ The corresponding H&E histopathological images were obtained from The Cancer Imaging Archive (TCIA).^3^ A total of 439 GBM patients were selected from TCGA based on the completeness of clinical records and image availability of high-quality histopathological images in TCIA, excluding cases with incomplete data. All included patients had corresponding genomic, transcriptomic, and proteomic data for a comprehensive multi-omics analysis. The GBM tissue microarrays (TMA) of 67 patients with clinical and follow-up data were purchased from Shanghai Outdo Biotech Co., Ltd. (Shanghai, China). Clinical information of patients involved in TMA and TCGA cohorts is provided in Supplementary materials 2, 3.

### 2.2 Image processing and feature extraction

To extract the quantitative features from whole-slide histopathological images, we applied the Openslide Python library (28) to segment the images into 1,000 × 1,000 pixel sub-images. Furthermore we randomly selected 50 sub-images on behalf of each patient to minimize selection bias and reduce computational load. Image feature extraction was conducted by CellProfiler (29),^4^ an open-source tool for biological-image analysis. The H&E-stained images were converted to grayscale for the extraction of features, which can be specifically categorized into 10 aspects including correlation, image area occupied, image granularity, image intensity, image quality, object intensity, object neighbors, object radial distribution, object size shape and texture. In particular, the textural features were calculated by CellProfiler to quantitatively present the perceived textures of histopathological images, thereby measuring the extent and nature of textures within objects in grayscale images. Through automatic identification and segmentation, these quantitative features objectively interpret the size, shape, spatial distribution, the texture of nucleus and the relationship of pixel intensities, etc. Afterwards, each sub-image was screened to exclude irrelevant features. Eventually, a total of 550 image features were extracted, with the average feature values of 50 representative sub-images of each slide calculated for subsequent analysis.

### 2.3 Statistical analysis

#### 2.3.1 Mutations and subtypes prediction

**Figure 2:**
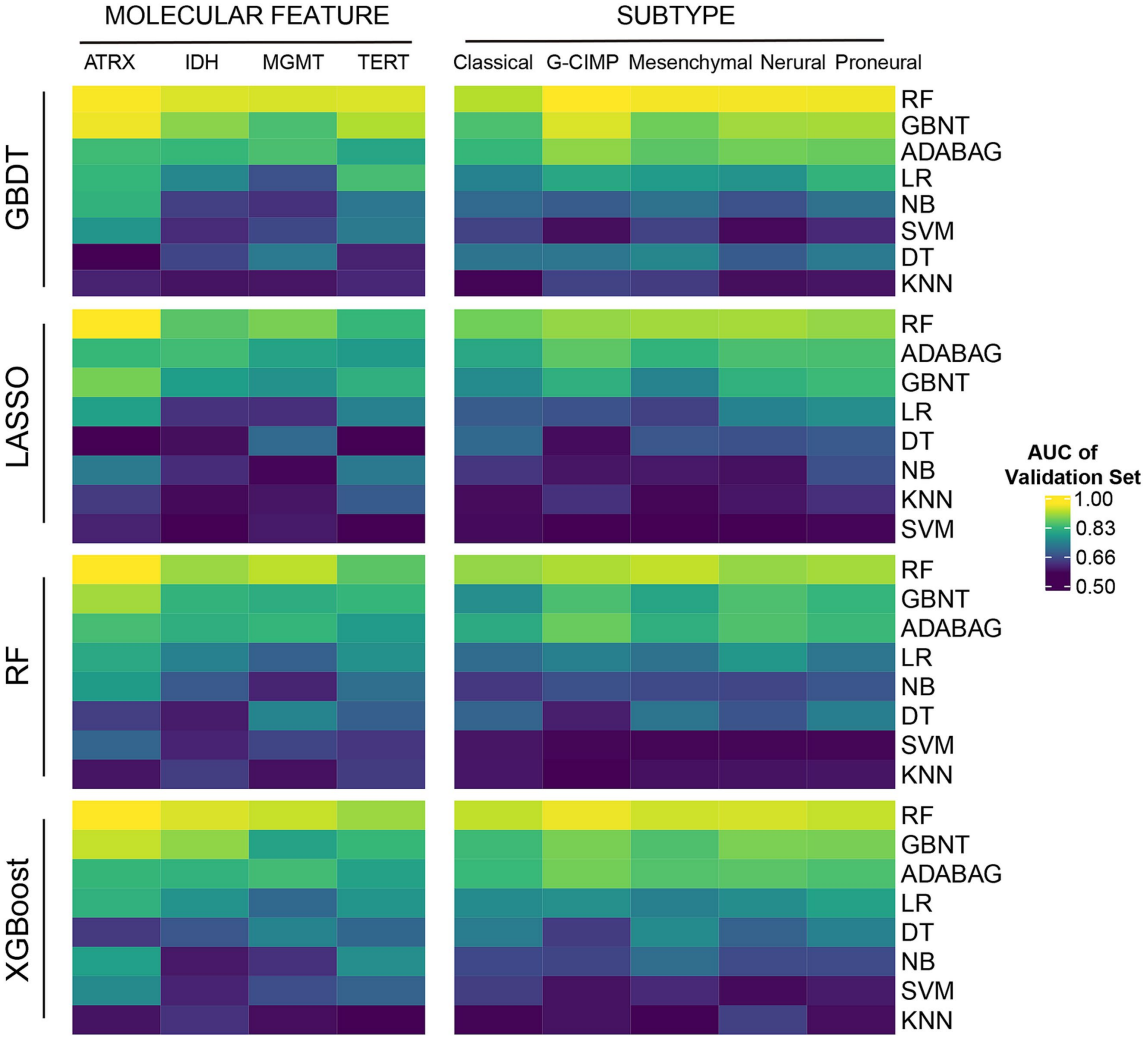
The predictive power of HIF in molecular features. Four machine learning algorithms (GBDT, LASSO, RF, and XGBoost) were applied for feature selection. Eight machine learning classifiers (RF, GBDT, Addaboost (ADABAG), LR, DT, SVM, NB, and KNN) were applied for molecular feature classification.

Initially, we randomly assigned the GBM samples into a training set and a test set by a ratio of 1:1 using R package “randomizr.” In order to reduce overfitting caused by the large number of features, we initially employed four machine learning algorithms for feature selection to extract the most informative histopathological image features (HIFs), including least absolute shrinkage and selection operator (LASSO) (30), random forest (RF) (31), gradient boosting decision tree (GBDT) (32), and extreme gradient boosting (XGBoost) (33). Subsequently, we evaluated eight classifiers including RF, GBDT, adaptive boosting (AdaBoost) (34), logistic regression (LR) (34), decision tree (DT) (35), support vector machine (SVM) (36), naive Bayesian (NB) (37) and K-nearest neighbor (KNN) (38) to determine the optimal classification algorithm through the prediction of frequent somatic mutations (i.e., ATRX, IDH, MGMT, and TERT) and molecular subtypes defined by transcription profiles and epigenetics (i.e., classical, mesenchymal, neural, proneural, and G-CIMP) based on the selected imaging features and evaluated with 5-fold cross-validation. By applying multiple approaches, we intended to verify the feasibility and stability of the method in different algorithms. Based on the test set, the performances of trained classifiers were validated and compared respectively, among which RF demonstrated the highest predictive accuracy, as evidenced in Supplementary material 1 and Figure 2.

#### 2.3.2 Survival analysis

For survival analysis, we divided patients in the training cohort into two groups based on the median value of individual HIFs, which was used for Kaplan–Meier survival analysis and log-rank test to compare overall survival (OS) between high-risk and low-risk groups, with *p* < 0.05 considered statistically significant. Univariate Cox regression was conducted based on all HIFs as continuous variables to determine the hazard ratio (HR) and 95% confidence interval (CI) and identify features significantly associated with overall survival.

#### 2.3.3 Data pre-processing and feature selection

To synthetically evaluate the prognostic value of various omics data types, we included independent omics data (HIF, genomics, transcriptomics and proteomics) and integration of multiple features (HIF + genomics, HIF + transcriptomics, HIF + proteomics and HIF + omics) for further analysis. Patients were randomly distributed into training and validation sets on a ratio of 1:1, ensuring a balanced subset size for model training and independent evaluation to assess generalizability. In the training set, we first included the 100 most frequent somatic mutations to reduce the dimensionality in genomics profile for subsequent analyses. Patients with an overall survival (OS) of over 60 months were categorized into the long-term survival group, while those with an OS of 1–12 months were placed in the short-term survival group. Differentially expressed genes (DEGs) between the two groups were conducted using the limma package in R, and the top 100 significant DEGs were used for survival prediction. Additionally, Metascape^5^ was employed for enrichment analysis based on the genomic profile.

#### 2.3.4 Prognostic models construction and validation

Based on the training set, we employed the random forest (RF) algorithm with 1,000 decision trees and 5-fold cross-validation to construct prognostic models via R randomForestSRC package. The RF algorithm is a dimension reduction method that has preferable performance in accessing vast amounts of input data and gives estimates of the importance of variables. It can also conduct internal unbiased estimates of the generalization error and improve model accuracy. Meanwhile, the RF includes its own regularization through tree pruning and ensemble learning. Furthermore, we performed model validation based on the validation set through the estimation of the AUC value of time-dependent ROC. Patients were then assigned to high-risk group and low-risk group in line with the median value of risk score computed by different models. Kaplan–Meier analysis and log-rank test were performed between the groups to evaluate the prediction capacity. Moreover, we carried out the decision curve analysis (DCA) based on validation set to compare the net benefit under a range of threshold probabilities of each model.

### 2.4 Selection of prognosis-related histopathological image features

Two machine learning methods including least absolute shrinkage and selection operator Cox (LASSO-Cox) regression (R package “glmnet”) and support vector machines-recursive feature elimination (SVM-RFE) (R package “e1071”) were performed independently to identify potential informative image features related to prognostic prediction. LASSO-Cox regression applies L1 regularization, effectively reducing multicollinearity, selecting the most survival-associated features and mitigating overfitting by shrinking less relevant coefficients to zero (39). The SVM model can classify data points by maximizing the distance of the hyperplane with high accuracy, thus identifying predictive models or classifiers. SVM-RFE is a feature selection algorithm according to recursive feature deletion sequences with maximum interval principle. It ranks features based on their contribution to classification performance, iteratively eliminating the least informative ones. The integration of LASSO-Cox and SVM-RFE has been demonstrated to improve the model’s generalizability and predictive performance by reducing overfitting and enhancing feature selection reliability (40, 41). Eventually, the features within the intersection of the results by two algorithms were identified as the prognostic histopathological image features (PHIF).

### 2.5 Gene co-expression network analysis

We performed weighted gene co-expression network analysis (WGCNA) based on training set to investigate the association of the prognostic histopathological image features and corresponding gene expression, aiming to further understand the upstream biological mechanisms. WGCNA (42) has been applied to identify modules of genes with highly correlated expression by analyzing the connections between corresponding genes and converting the expression profile into the weighted network. Co-expressed gene networks may facilitate the identification of underlying biological processes, candidate biomarkers and certain clinical traits. Additionally, we applied Metascape for enrichment analysis to estimate the interlinkage between key modules.

## 3 Results

### 3.1 Prediction performance of HIF on somatic mutations and molecular subtypes

In total we included 439 GBM patients with the matched information of histopathological images and other omics from TCGA portal. To minimize overfitting caused by high-dimensional image features, we initially employed XGBoost, GBDT, LASSO, and RF for feature selection and extracted 550 histopathological image features (HIFs) out of the segmented tumor tissue images. Subsequently, to evaluate the clinical practicability of the 550 HIFs, we employed eight algorithms (RF, GBDT, AdaBoost, LR, DT, SVM, NB, and KNN) as classifiers in predicting four common somatic mutations (ATRX, IDH, MGMT, and TERT) and five RNA-based molecular subtypes (classical, mesenchymal, neural, proneural, and G-CIMP). We systematically compared the predictive performances of all classifiers across multiple molecular features, and RF consistently achieved the highest predictive accuracy among the eight classifiers, independent of the feature selection method used. The AUC values for RF models showed superior classification ability across all tested molecular characteristics as shown in Figure 2 and Supplementary material 1. Therefore, we selected RF as a robustly performed algorithm for subsequent prognostic model construction. Additionally, the HIF models validated by GBDT and AdaBoost (ADABAG) also achieved a relatively accurate classification effect under different feature screening methods, which indicates the clinical practicability of HIFs in distinguishing the somatic mutations and molecular subtypes of GBM.

### 3.2 Prognostic value evaluation of histopathological image features

**Figure 3:**
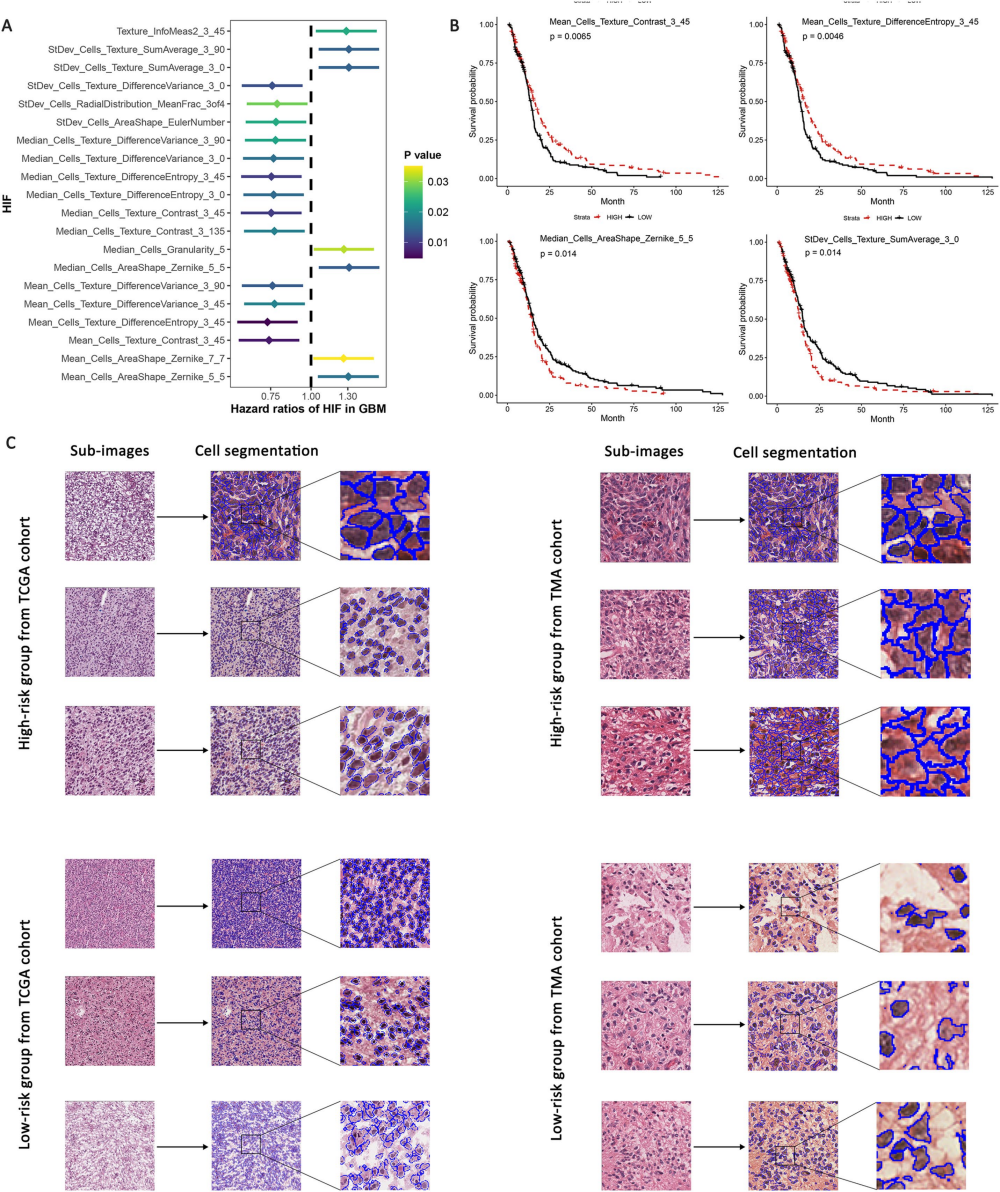
Univariate survival analyses based on HIF. GBM patients were assigned into high-risk and low-risk group according to the median value of each feature. **(A)** Hazard ratio of survival difference between two groups in univariate Cox regression. **(B)** Kaplan–Meier curves for groups with high-value and low-value “Median_Cells_AreaShape_Zernike_5_5,” “Mean_Cells_Texture_Contrast_3_45,” “Mean_Cells_Texture_DifferenceEntropy_3_45” and “StDev_Cells_Texture_SumAverage_3_0.” **(C)** Representative sub-images of high-risk and low-risk groups in both TCGA and TMA validation cohorts.

To assess the correlation between histopathological image features (HIFs) and the prognosis of GBM patients, we conducted survival analyses based on individual HIFs. We first assigned the patients into two groups in line with the median value of each HIF (higher than median vs. lower than median) for survival analyses. Afterwards, we carried out univariate Cox analyses based on all HIFs to identify protective prognostic imaging factors, and the top 20 features significantly correlated with the overall survival (OS) was demonstrated in Figure 3A. The four most significant HIFs, with the smallest *p*-value included one Zernike shape feature (Median_Cells_AreaShape_Zernike_5_5) and three cell texture features (Mean_Cells_Texture_Contrast_3_45, Mean_Cells_Texture_DifferenceEntropy_3_45 and StDev_Cells_Texture_SumAverage_3_0). In particular, Zernike features are a series of 30 shape features based on Zernike polynomials, ranging from order 0 to order 9, which have been frequently extracted for representing the shape parameters in cell nucleus. Cell texture features quantify the correlations between nearby pixels in the regions of interest, which suggests that the global modes of cell nuclei and cytoplasm are all related to clinical survival outcomes. The Kaplan–Meier survival curves of four image features indicated significant differences between groups with high-value and low-value features, demonstrating the feasibility of HIFs in predicting the survival of GBM patients (Figure 3B).

Additionally, according to the expression level of the four predictive features mentioned above, we evaluated the sub-images of high-expressed and low-expressed prognostic features. We utilized TCGA internal validation and TMA external validation cohorts to assess the robustness of the predictive models and reduce the potential overfitting to the specific characteristics of the initial dataset. These validation steps serve as important safeguards against overfitting and bias, which enhances the reliability of our models across diverse datasets. The patients were identified as high-risk and low-risk groups based on the median value of risk scores, and the representative histopathological sub-images showed visible differences in TCGA and TMA external validation cohorts (Figure 3C). The image processing involving cell recognition and segmentation was conducted by CellProfiler, and different cell types were also outlined.

### 3.3 Integrated prognostic model of histopathological image features and genomics

**Figure 4:**
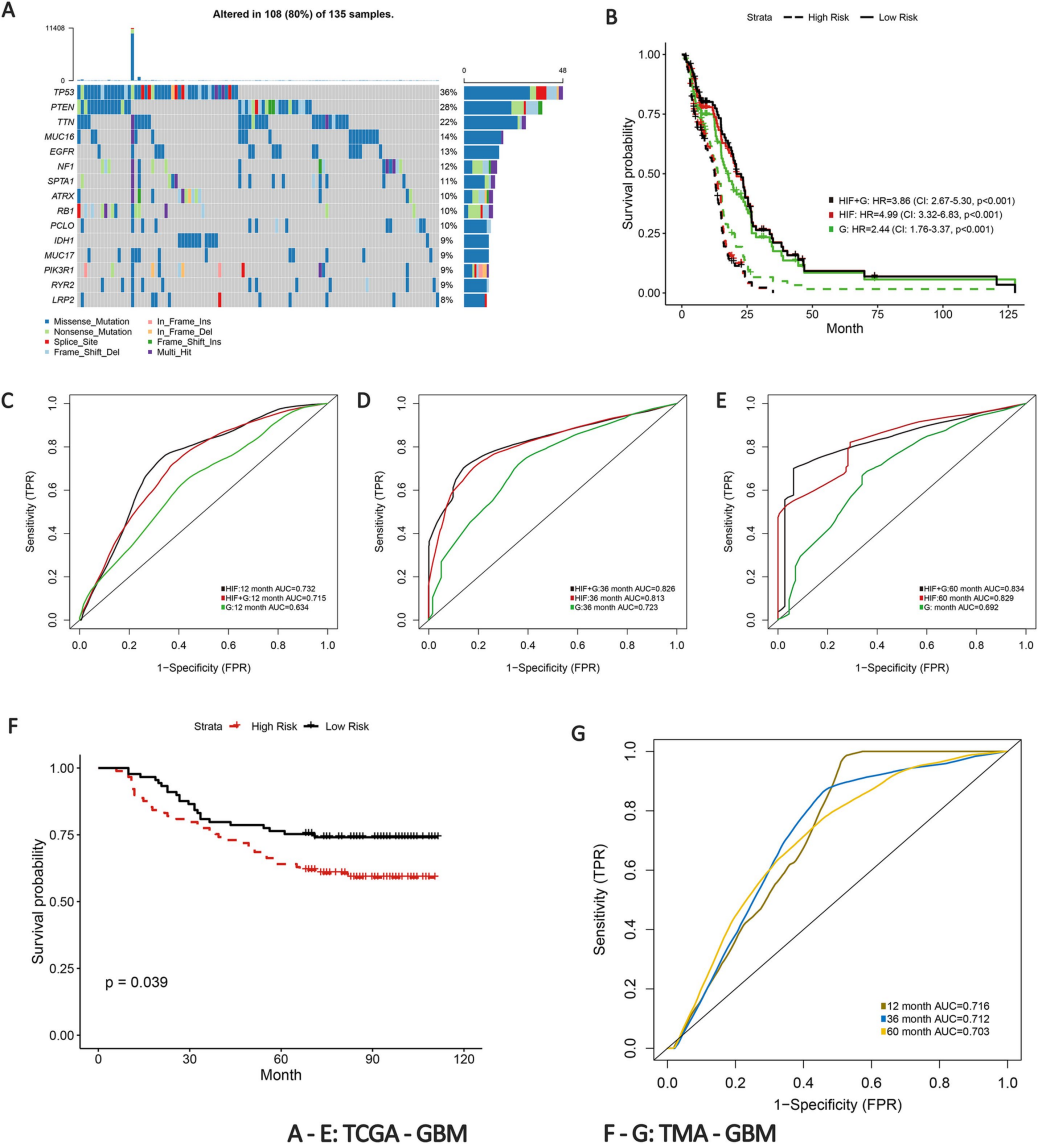
Prognostic models integrating HIF and genomics. **(A)** The waterfall plot of the top 15 most common somatic mutations in training set. **(B)** Kaplan–Meier curves of histopathological image features model (HIF), genomics model (G) and integrative histopathology + genomics model (HIF + G) in the validation set. **(C–E)** The **(C)** 1-year, **(D)** 3-year, and **(E)** 5-year area under the time-dependent receiver operating curve (AUC) of the three prognostic models in the validation set. **(F)** Kaplan–Meier curves of high-risk group and low-risk group in the TMA external validation cohort. **(G)** Time-dependent ROC of 1-year, 3-year, and 5-year OS in the TMA external validation cohort.

To develop a more accurate predictive model for overall survival (OS) in GBM patients, we estimated the prognostic value of genetic profiles and further incorporated the HIFs with genomics data. Patients were randomly assigned into training (*n* = 136) and validation (*n* = 135) sets. To enhance the stability of the measurement, we estimated the mutation status of genes in training set and included the 100 most common somatic mutations in the prognostic model to reduce the dimensionality of the genomics data. The top 15 genes with the most frequent alterations are presented in Figure 4A. Based on the HIFs and 100 mutations we constructed prognosis-relate models in the training set. We applied time-dependent ROC in the validation set since it is more appropriate to represent time-to-event outcomes in the prognostic models compared to the classical ROC curve analysis approach (43). As illustrated in Figures 4C–E, the AUCs for histopathological image features (HIF) model exceeded those of genomics (G) model in 1-year (0.715 vs. 0.634), 3-year (0.813 vs. 0.723) and 5-year (0.829 vs. 0.692) respectively. Moreover, the integrated model of HIF and genomics (HIF + G) reached a better predictive capacity in 3-year and 5-year (AUC = 0.826 and 0.834) than the former two single-omics models. According to the median value of risk score acquired from each model, the patients were then divided into high-risk and low-risk groups. The HIF model and integrative model (HIF + G) showed more accurate prognostic performance (HR = 3.86, 95%CI: 2.67–5.30, *p* < 0.001, Figure 4) as depicted in Kaplan–Meier curves (Figure 4B).

To further validate the predictive power of the prognostic model, we implemented an external verification using the TMA-GBM cohort. Patients in the external validation set were also divided into high-risk and low-risk groups according to the median risk score. The Kaplan–Meier survival curve revealed a significant difference in survival probability between the groups (*p* = 0.039, Figure 4F). The 1-year, 3-year and 5-year AUCs of time-dependent ROC were 0.716, 0.712, and 0.703, respectively (Figure 4G). The results thus verified the prognostic capacity of the HIFs in GBM patients.

### 3.4 Integrated prognostic model of HIF and transcriptomics

**Figure 5:**
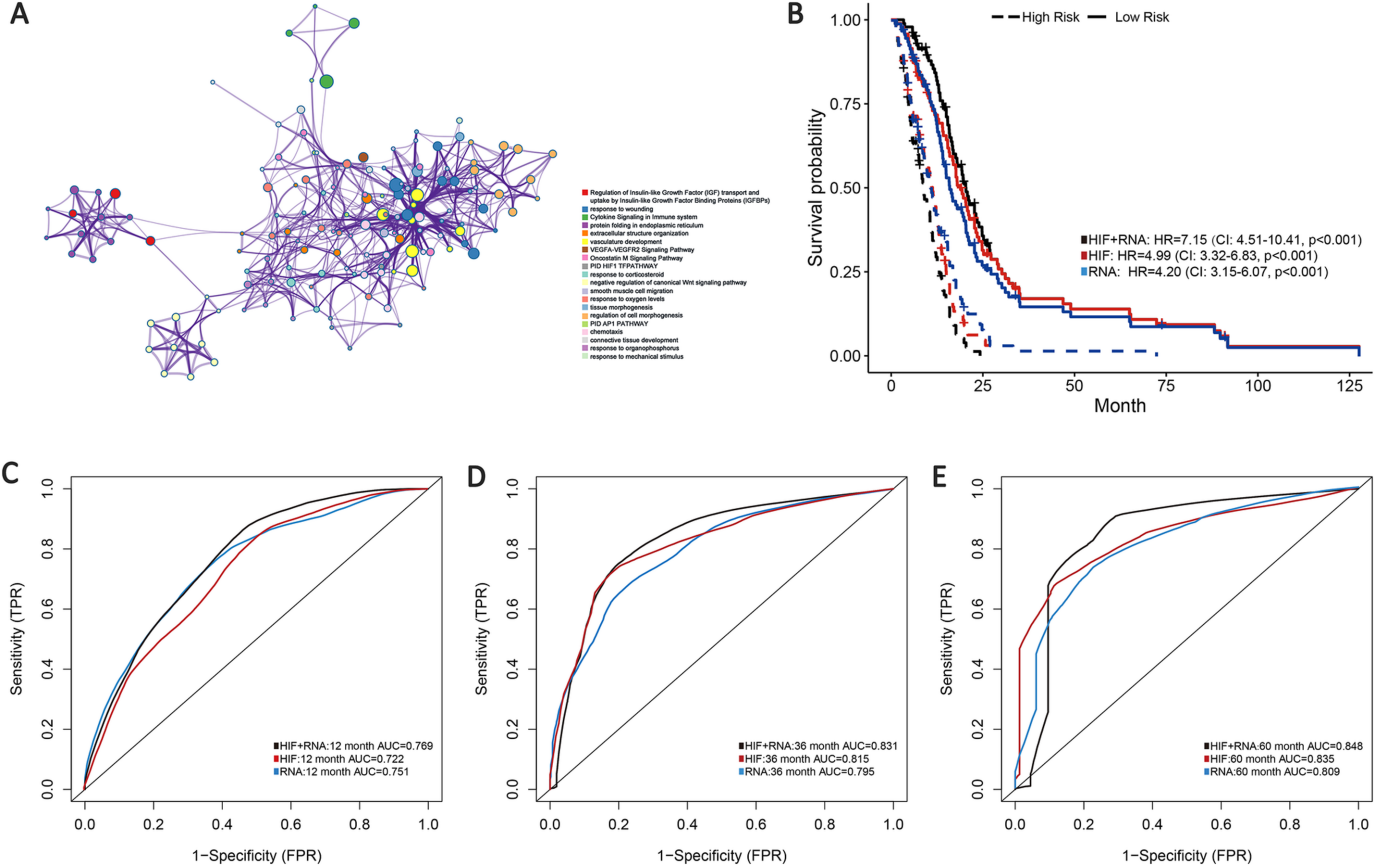
Prognostic models integrating HIF and transcriptomics (RNA). **(A)** Metascape enrichment network visualization cluster of genes and associated biological pathways based on training set. Each circled node represents a term and each color represents its cluster identification, showing the intra-cluster and inter-cluster similarities of enriched terms. **(B)** Kaplan–Meier curves of prognostic models (HIF, RNA, and HIF + RNA) in the validation set. **(C–E)**. The **(C)** 1-year, **(D)** 3-year and **(E)** 5-year AUCs of the three prognostic models in the validation set.

Transcriptomics can serve as an approach for a comprehensive understanding of the interconnection between the genome, proteome, and cellular phenotype by analyzing the RNA transcripts that reflect the underlying genotype. Based on the training set, we involved 100 whole expressed mRNA genes to decrease the dimensionality and further build the transcriptomics predictive model of OS. The patients were categorized into short-term group (deceased, 12 months ≥ OS ≥1 month) and long-term group (OS ≥60 months) according to the clinical survival status (4, 5, 7, 8). In addition, we applied Metascape for pathways enrichment in the short-term survival group based on the mRNA sequencing data (Figure 5A). Regulation of insulin-like growth factor (IGF) transport and uptake by insulin-like growth factor binding proteins (IGFBPs) has been proven to modulate essential cellular processes and be implicated in certain disorders including malignant, metabolic and immune diseases (44, 45). Previous studies have reported the potential effect of IGF in biological processes associated with tumor growth and invasion inhibition in GBM (46), which may suggest a new effective target for anti-cancer treatment strategies.

As demonstrated in the validation set, the transcriptomics model (RNA) displayed a good predictive performance for OS (1-year AUC = 0.751, 3-year AUC = 0.795 and 5-year AUC = 0.809), which were about equal to the HIF model (1-year AUC = 0.722, 3-year AUC = 0.815 and 5-year AUC = 0.835). Furthermore, we incorporated the transcriptomics and image features as the integrated model (HIF + RNA), which achieved the highest accuracy with the 1-year, 3-year and 5-year AUC increased to 0.769, 0.831 and 0.848 (Figures 5C–E). Additionally, Kaplan–Meier survival analyses also revealed significant differences in survival outcomes between the two groups, with the integrative HIF + RNA model presenting the most notable prognostic value (HR = 7.15, 95%CI: 4.51–10.41, *p* < 0.001, Figure 5B).

### 3.5 Integrated prognostic model of HIF and proteomics

**Figure 6:**
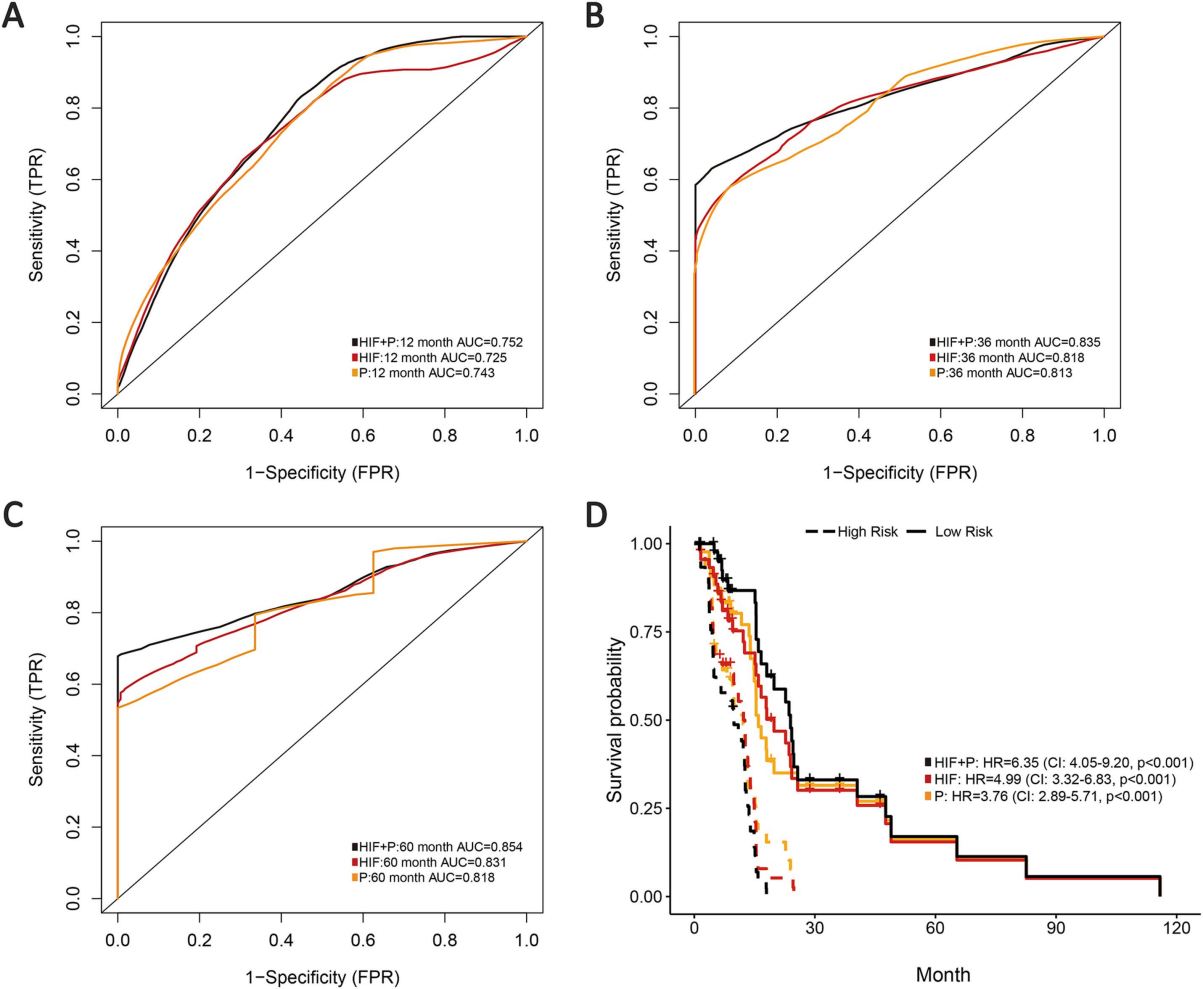
Prognostic models integrating HIF with proteomics (P). **(A–C)** The **(A)** 1-year, **(B)** 3-year and **(C)** 5-year AUCs of the three prognostic models (HIF, P and HIF + P) in the validation set. **(D)** Kaplan–Meier curves of the three prognostic models in the validation set.

To improve the prognostic prediction of GBM we also incorporated proteomics profile from TCPA portal for further analysis through the reverse phase protein array (RPPA), a high-throughput proteomics method that can assess protein expression and activation states in abundant samples using small amounts of material. In total we involved 179 eligible protein profiles in the proteomics model based on the validation set. The integration of image features and proteomics features (HIF + P) achieved the highest AUCs in 1-year, 3-year and 5-year compared with the proteomics model (0.752 vs. 0.743, 0.835 vs. 0.813, 0.854 vs. 0.818) or the HIF model alone (Figures 6A–C). As shown in the survival analyses, patients in the high-risk group were significantly related to poor OS, and the integrated model (HIF + P) attained the best performance in prognosis prediction among the three models (HR = 6.35, 95%CI: 4.05–9.20, *p* < 0.001, Figure 6D).

### 3.6 Integrated multi-omics features for survival prediction

**Figure 7:**
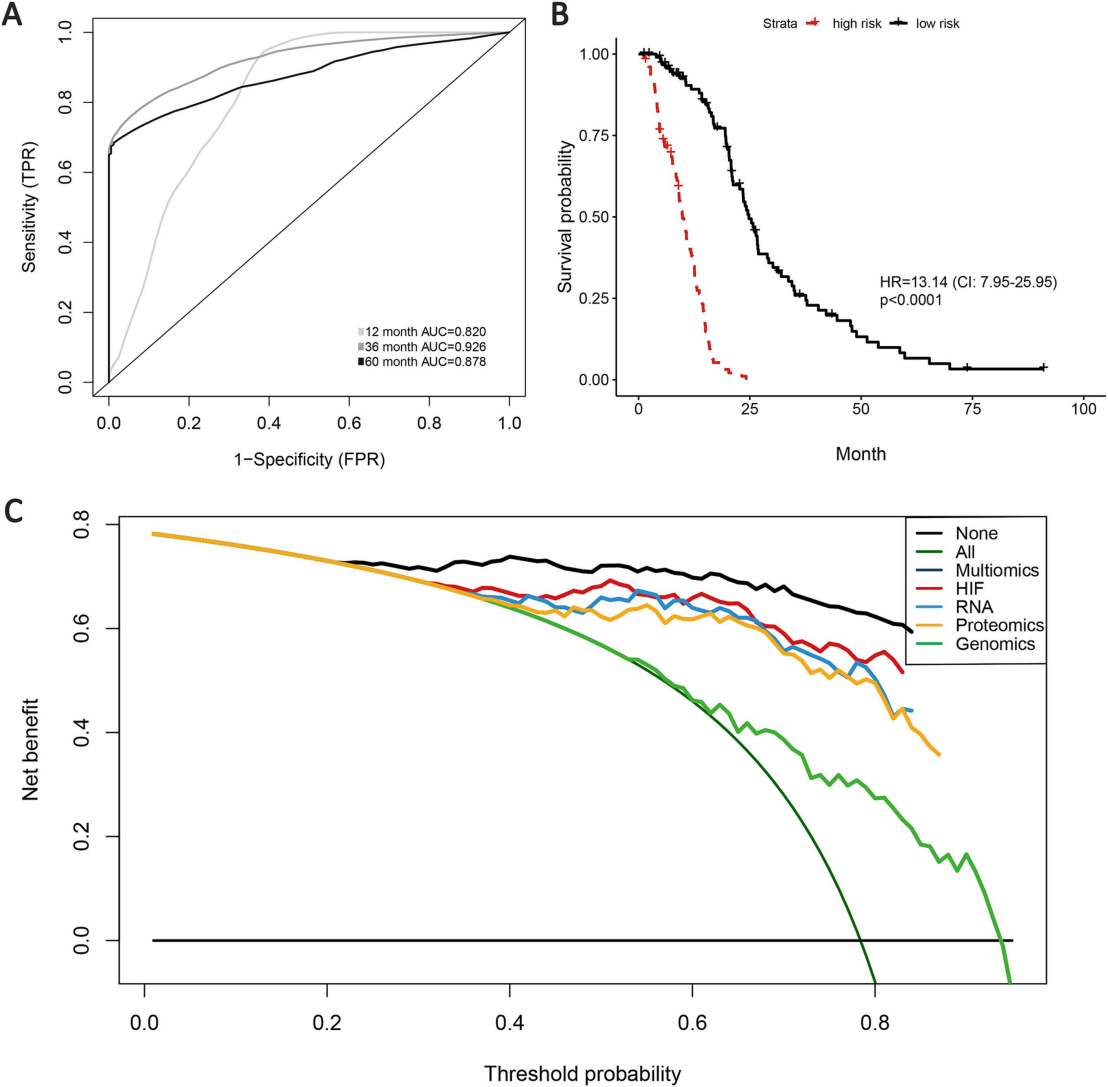
Prognostic models of survival integrating HIF and multiple omics features. **(A)** AUCs of multi-omics model in the validation set. Kaplan–Meier curve of multi-omics model (integrating HIF, radiomics, genomics, transcriptomics, proteomics) in the validation set. **(B)** Decision curves analysis for different models in the validation set. **(C)** The gray oblique line represented the net benefit of intervention for all patients, while the horizontal line represented the net benefit of no intervention. The multi-omics model achieved higher net benefit than single-omics models across the major range of threshold probability.

According to the previous analyses, the histopathological image features have presented certain effectiveness in prognostic prediction for GBM patients, and histopathology + omics models have also indicated enhancement in predictive performance and accuracy than the single-omics models. Therefore, we expect to explore the prognostic capacity of a multi-omics predictive model incorporating all the omics features (HIF, genomics, transcriptomics, and proteomics). Based on the validation set, the multi-omics model achieved a 1-year AUC of 0.820, 3-year AUC of 0.926 and 5-year AUC of 0.878, representing an improvement over the HIF + genomics, HIF + transcriptomics and HIF + proteomics models (Figure 7A). Kaplan–Meier survival analysis illustrated a significant difference in survival between high-risk and low-risk groups (HR = 13.14, 95% CI: 7.95–25.95, *p* < 0.001, Figure 7B). Furthermore, the multi-omics model demonstrated superior net benefit in survival prediction compared to the other models (Figure 7C).

**Figure 8:**
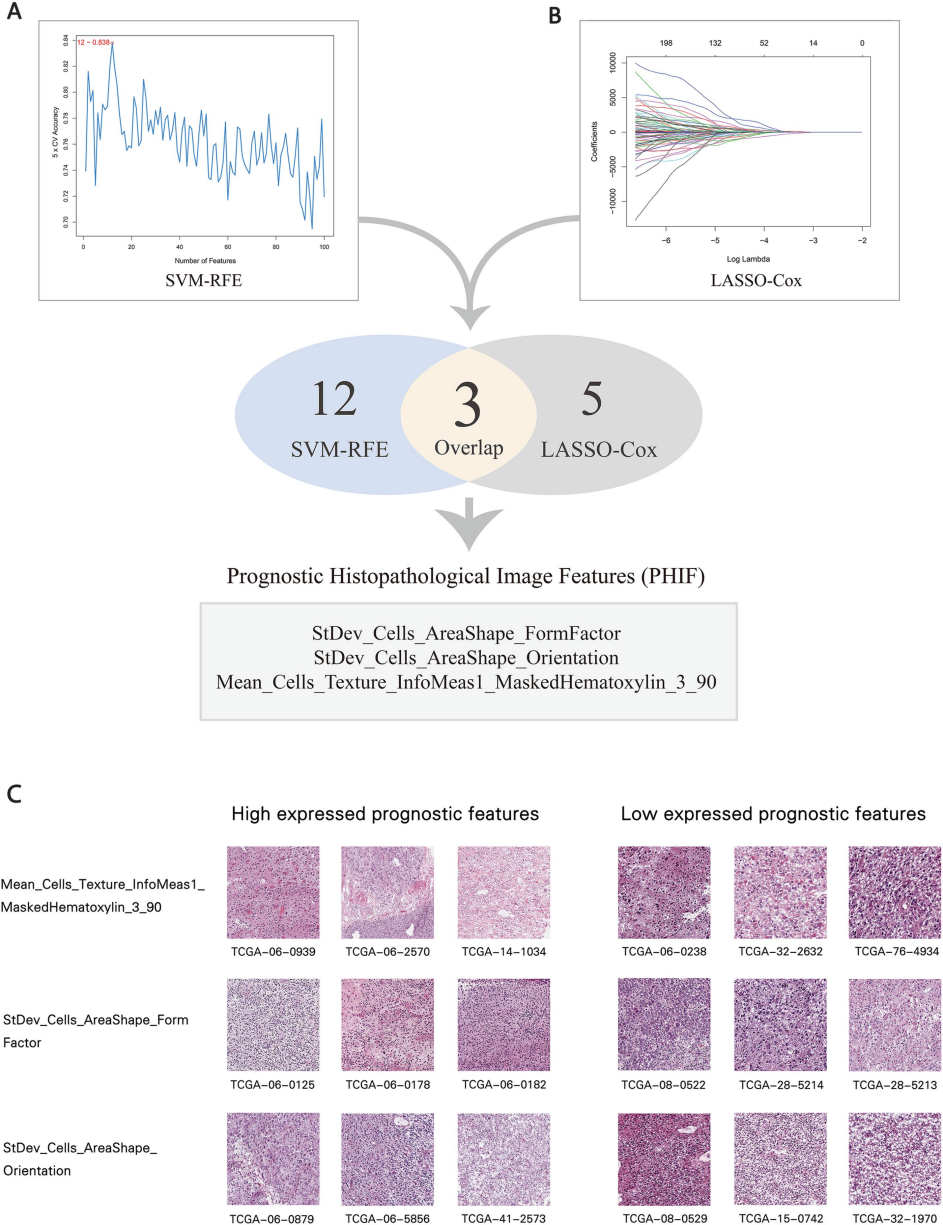
Selection of prognostic histopathological image features (PHIF). **(A)** Twelve image features were selected by SVM-RFE. **(B)** Five image features were selected by LASSO-COX regression model. Three image features within the overlap were defined as PHIF. Three image features within the overlap were defined as PHIF. **(C)** Representative sub-images of patients with high expressed and low expressed PHIF. The groups were defined by the median value of each image feature.

In order to identify the histopathological image features with higher prognostic value for OS, LASSO-Cox regression and SVM-RFE were performed independently. These combined approaches help mitigate the risk of overfitting and ensure the robustness of selected features across different selection frameworks. Previous studies (39–41) have demonstrated that the combination of LASSO and SVM-RFE enhances the reliability of prognostic feature identification in cancer research. A total of five imaging features involved in prognosis were selected via LASSO-Cox regression model, and SVM-RFE selected 12 imaging features with the most significant predictive ability. Ultimately, three overlapped features were identified as prognostic histopathological image features (PHIF), including StDev_Cells_AreaShape_FormFactor, StDev_Cells_AreaShape_Orientation and Mean_Cells_Texture_InfoMeas1_MaskedHematoxylin_3_90 (Figures 8A,B). Representative sub-images and detailed information of patients with high expressed and low expressed PHIF were displayed in Figure 8C and Supplementary material 4.

**Figure 9:**
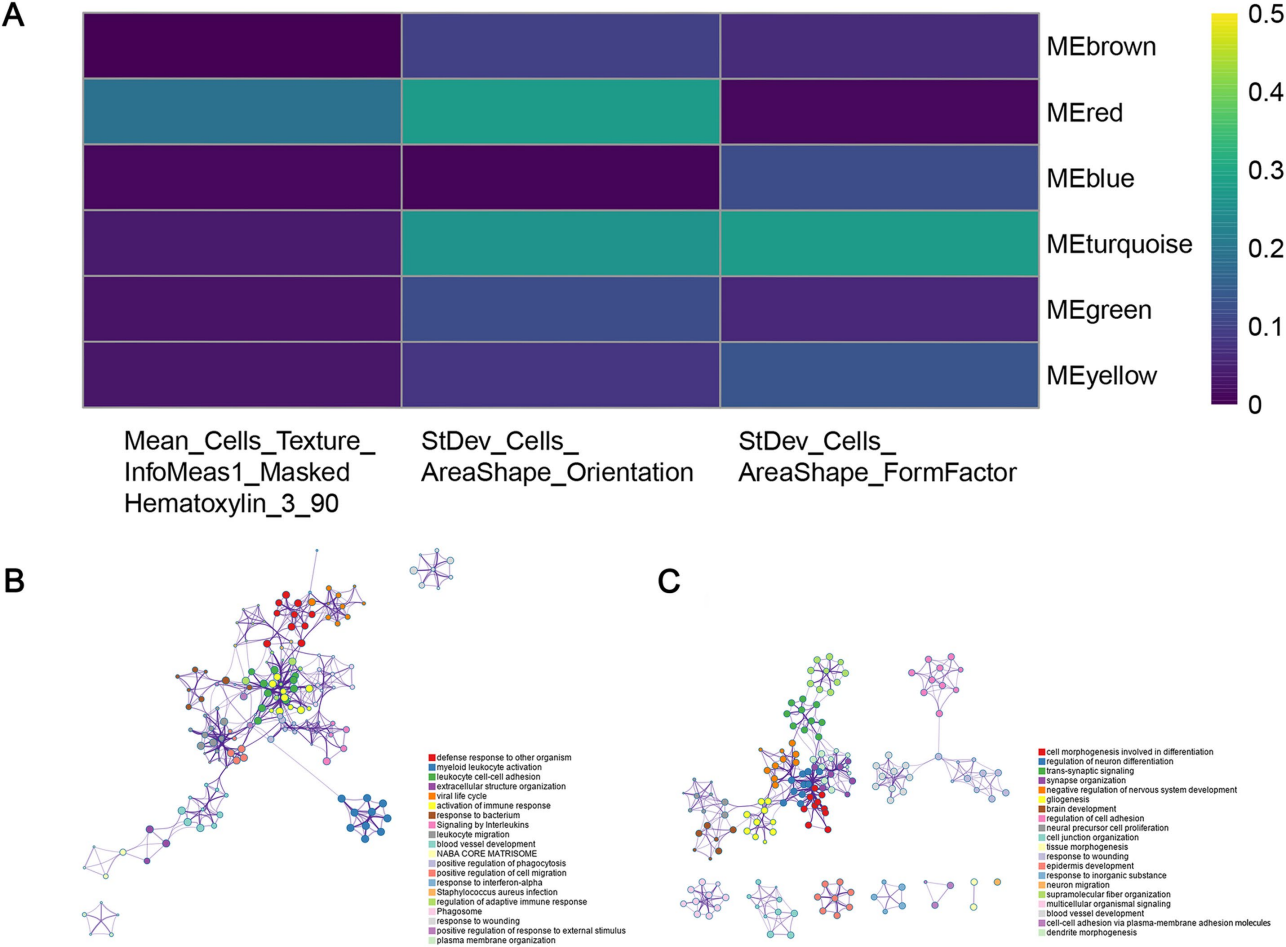
Identification of co-expressed gene modules. **(A)** Heatmap of the relationship between gene modules and prognostic histopathological image features (PHIF) through WGCNA. The red module and turquoise module showed the most significant correlation. **(B)** Metascape enrichment network of genes in the red module. Each circled node represents a term and each color represents its cluster identification, showing the intra-cluster and inter-cluster similarities of enriched terms. **(C)** Metascape enrichment network of genes in the turquoise module.

To explore the upstream genetic mechanisms, we employed WGCNA to construct a gene co-expression network in the training set and identify the gene clusters significantly correlated with the PHIF in GBM samples. Module-trait correlation analysis showed that the red module (219 genes) and turquoise module (868 genes) were significantly associated with the three prognostic image features of GBM among the six identified gene co-expression modules (Figure 9A). Therefore, we defined the red and turquoise module as the key modules of significant prognostic relevance for subsequent research.

Subsequently, we performed an enrichment analysis to explain the biological interpretations of the gene expression profile in the two modules. Genes in the red module were significantly related to several biological processes and pathways such as defense response to other organism, myeloid leukocyte activation, leukocyte cell–cell adhesion, activation of immune response and response to bacterium (Figure 9B). The results indicated that these genes may be involved in immune function, a crucial aspect of tumor immunology, which plays an important role in tumor initiation and progression. The genes in the turquoise module were primarily enriched in categories related to cell morphogenesis involved in differentiation, regulation of neuron differentiation and nervous system development, synapse organization and signaling (Figure 9C). These findings implied that turquoise module genes may have potential association with central nervous system pathways and cerebral function, which may correspond to tumorigenesis and progression in GBM.

## 4 Discussion

In this study, we extracted quantitative image features from histopathological images of GBM patients, and subsequently constructed machine learning classifiers based on the HIFs to discriminate the common molecular features of GBM. A predictive model incorporating HIFs was established in the training set, with its prognostic validity subsequently verified in both internal and external validation cohorts. The results demonstrated the prognostic robustness of the predictive model. To enhance the predictive performance, comprehensive prognostic models were built by integrating HIFs with multi-omics data. Based on machine learning approaches, we selected prognostic histopathological image features (PHIF) and identified gene modules most strongly correlated with PHIF through bioinformatics techniques. Notably, the predictive power of OS in patients was significantly enhanced in multi-omics models compared with the single-omics models, suggesting that this approach may be promising for risk stratification and individualized treatments for GBM patients.

Based on histopathological image features, we performed the prediction of the common somatic mutations (ATRX, IDH, and TERT) and methylation (MGMT) in GBM through combinations of eight independent machine learning algorithms. IDH mutations, which occur in approximately 12% of GBM cases, are a well-established prognostic marker associated with prolonged OS (47). The mutation can induce downstream effects on cellular metabolism and epigenetic regulation (48). Previous studies have reported the predictive value of MRI radiomics models for identifying IDH1 mutations in GBM (49, 50), as well as the characterization of core signaling pathways in IDH wild-type tumors (51). The prediction ability of histopathological image features in IDH mutation has not been widely explored, while it may represent an important avenue for further research in prognostic evaluation and targeted therapies for GBM. MGMT methylation status and TERT promoter mutations have also been recognized as powerful diagnostic and prognostic indicators in GBM (2, 52). Meanwhile, we also conducted the prediction of four mRNA-based molecular subtypes (classical, mesenchymal, neural, proneural) and the G-CIMP methylator phenotype. The prognostic significance of G-CIMP+ subsets among glioma types has been investigated in previous studies (53, 54). For instance, 1p/19q codeletion and MGMT promoter methylation may act as therapeutic predictive markers in GBM (55). Our random forest predictive model based on HIFs exhibited certain accuracy and effectiveness in predicting GBM molecular characteristics, which may contribute to improving current clinical examinations and diagnostic practices.

Subsequently, we constructed prognostic models through random forest algorithm based on single-omics and integrated multi-omics data. Image features of histopathology tissue slides can infer morphological changes in tumor cells and microenvironment, which have proven valuable in identifying pathology biomarkers and predicting clinical outcomes through machine learning techniques (56–58). A fair number of computational histopathologic models have also been applied in the prognostic prediction of diseases such as breast (59), lung (60) and colorectal cancers (61). Consistent with previous studies, the image features with significant prognostic power of OS we selected primarily pertained to Zernike and cell texture (i.e., contrast, sum average, and difference entropy). Zernike shape features in nuclei and cytoplasm are extracted frequently to identify long and short term survival (62). In addition, the texture features are frequently used to represent the distribution and variation of pixel intensity, as well as the relationship between pairs with different intensity values in the regions of interest. While many studies have established prognostic modules based on single-omics data source or combination of quantitative histopathological image features and genomics features (21, 53), our study focused on a more comprehensive evaluation of image features to provide additional prognostic efficiency and precision of the prognostic model. By integrating HIFs with genomics, transcriptomics and proteomics data, we developed a multi-omics model incorporating all features, which eventually achieved superior prediction performance compared to other models. Additionally, we further proposed external validation by involving an extra TMA cohort, further supporting the robustness and generalizability of our findings.

An intriguing observation in our study was that the model based solely on HIFs slightly outperformed the combined HIF and genomics (HIF + G) model in terms of predictive performance, as shown in Figure 4B. This unexpected finding prompted further reflection on the interaction between histopathological and genomic data in prognostic modeling. One possible explanation lies in feature redundancy and confounding effects that HIFs inherently capture tumor morphological and microstructural features, which may already correlate with patient prognosis. The addition of genomic features that provide overlapping or weakly correlated prognostic signals may introduce noise rather than improving predictive accuracy. This aligns with established principles in machine learning, where the mere inclusion of additional variables does not necessarily enhance model performance; instead, feature interactions must be carefully managed to avoid confounding effects. Moreover, the non-linearity between histopathological and genomic data may contribute to this outcome. While HIFs reflect macroscopic tumor morphology, genomic alterations influence prognosis through intricate molecular pathways that may not exhibit direct correlations with image-derived features. Traditional machine learning models may struggle to capture these complex interactions effectively, highlighting the need for alternative fusion strategies such as deep learning or graph neural networks to better integrate data from different modalities.

Despite the robust predictive power of HIFs alone, we emphasize the importance of multi-omics integration for comprehensive patient profiling. While the HIF + G model did not significantly outperform the HIF model alone, the incorporation of transcriptomic and proteomic data substantially improved the accuracy of our prognostic models. This suggests that multi-omics integration holds promise for enhancing model generalizability and robustness across diverse patient populations. Further optimization of feature selection and model refinement will be necessary to fully leverage the potential of multi-omics data.

Through SVM-RFE and LASSO-Cox regression machine learning algorithms, we identified three prognostic histopathological image features (PHIF) concerning cell morphology and texture. We also explored the upstream molecular mechanisms of these features by identifying relevant gene co-expression modules via weighted gene co-expression network analysis (WGCNA). Enrichment analysis of the red and turquoise gene modules demonstrated significant prognostic association with molecular pathways mainly involved in immune response, cell morphogenesis involved in differentiation, development and regulation of central nervous system function. For instance, leukocyte cell adhesion plays a crucial role in the progression and resolution of innate immunity (63). Myeloid leucocyte activation reveals exposure to activating factors and has been regarded as one of the major forces in immunosuppression in tumor progression (64). The genes enriched in cell morphogenesis related pathways might suggest the association with tumor angiogenesis and cell adhesion. In addition, regulation of neuron differentiation, trans-synaptic signaling and gliogenesis also suggest a close connection with biological processes in GBM development (65–67). The results may offer an opportunity to comprehend the association of histopathological image features and the upstream mechanisms of the oncogenesis and progression of GBM.

In conclusion, this study demonstrated the potential of histopathological image features in predicting molecular characteristics and classifying molecular subtypes. By integrating histopathological image features with multi-omics data, we developed comprehensive prognostic models and subsequently analyzed the associated upstream biological processes. The integrative multi-omics model has the potential to enhance prediction performance for OS with greater accuracy and robustness, thereby contributing to risk stratification, prognostic evaluation, and personalized treatment strategies for GBM patients.

However, several limitations should be addressed. Firstly, while the prognostic models were validated using an external TMA cohort to assess prediction stability, a larger-scale multi-center dataset is needed to enhance the applicability and reliability of our findings. Secondly, the genomic features of patients with intermediate survival (12–60 months) warrant further investigation, as they may provide additional insights into treatment response and prognostic markers. Additionally, discrepancies and potential biases in multi-omics data could impact the results. Future research should explore alternative data integration strategies to optimize the synergy between histopathology and molecular alterations. We also acknowledge the lack of unified visualization for all survival curves and model comparisons. Although constrained by computational limitations, we recognize the value of such visual summaries and are committed to improving model visualization and interpretability in future work, hoping to provide clearer insights for both clinical and research applications. Lastly, further clinical and experimental research is required to elucidate the molecular mechanisms underlying the relationship between histopathological image features and survival outcomes in GBM patients.

## Data Availability

The original contributions presented in the study are included in the article/Supplementary material, further inquiries can be directed to the corresponding authors.

## Footnotes

^1^ https://portal.gdc.cancer.gov

^2^ http://tcpaportal.org/tcpa/

^3^ http://www.cancerimagingarchive.net/

^4^ https://cellprofiler.org/

^5^ http://metascape.org

## Glossary

AdaBoost: Adaptive boosting
AUC: Area under the curve
CI: Confidence interval
DCA: Decision curve analysis
DEG: Differently expressed gene
DT: Decision tree
GBDT: Gradient boosting decision tree
GBM: Glioblastoma
HIF: Histopathological image features
HR: Hazard ratio
KNN: K-nearest neighbor
LASSO: Least absolute shrinkage and selection operator
LR: Logistic regression
NB: Naive Bayesian
OS: Overall survival
PHIF: Prognostic histopathological image features
RF: Random forest
ROC: Receiver operating characteristic
SVM: Support vector machine
TMA: Tissue microarrays
WGCNA: Weighted gene co-expression network analysis
XGBoost: Extreme gradient boosting
